# The Dyslexia Candidate Locus on 2p12 Is Associated with General Cognitive Ability and White Matter Structure

**DOI:** 10.1371/journal.pone.0050321

**Published:** 2012-11-28

**Authors:** Thomas S. Scerri, Fahimeh Darki, Dianne F. Newbury, Andrew J. O. Whitehouse, Myriam Peyrard-Janvid, Hans Matsson, Qi W. Ang, Craig E. Pennell, Susan Ring, John Stein, Andrew P. Morris, Anthony P. Monaco, Juha Kere, Joel B. Talcott, Torkel Klingberg, Silvia Paracchini

**Affiliations:** 1 Wellcome Trust Centre for Human Genetics, University of Oxford, Oxford, United Kingdom; 2 Department of Neuroscience, Karolinska Institutet, Stockholm, Sweden; 3 Telethon Institute for Child Health Research, Centre for Child Health Research, University of Western Australia, West Perth, Australia; 4 Department of Biosciences and Nutrition at Novum, Karolinska Institutet, Stockholm, Sweden; 5 School of Women's and Infants' Health, University of Western Australia, Crawley, Australia; 6 School of Social and Community Medicine, University of Bristol, Bristol, United Kingdom; 7 Department of Physiology, University of Oxford, Oxford, United Kingdom; 8 Molecular Medicine Research Programme, University of Helsinki, and Folkhälsan Institute of Genetics, Helsinki, Finland; 9 Science for Life Laboratory, Karolinska Institutet, Stockholm, Sweden; 10 Aston Brain Centre, School of Life and Health Sciences, Aston University, Birmingham, United Kingdom; 11 School of Medicine, University of St Andrews, St Andrews, United Kingdom; University of Cambridge, United Kingdom

## Abstract

Independent studies have shown that candidate genes for dyslexia and specific language impairment (SLI) impact upon reading/language-specific traits in the general population. To further explore the effect of disorder-associated genes on cognitive functions, we investigated whether they play a role in broader cognitive traits. We tested a panel of dyslexia and SLI genetic risk factors for association with two measures of general cognitive abilities, or IQ, (verbal and non-verbal) in the Avon Longitudinal Study of Parents and Children (ALSPAC) cohort (N>5,000). Only the *MRPL19/C2ORF3* locus showed statistically significant association (minimum P = 0.00009) which was further supported by independent replications following analysis in four other cohorts. In addition, a fifth independent sample showed association between the *MRPL19/C2ORF3* locus and white matter structure in the posterior part of the corpus callosum and cingulum, connecting large parts of the cortex in the parietal, occipital and temporal lobes. These findings suggest that this locus, originally identified as being associated with dyslexia, is likely to harbour genetic variants associated with general cognitive abilities by influencing white matter structure in localised neuronal regions.

## Introduction

Dyslexia (or reading disability, RD) and specific language impairment (SLI) are common neurodevelopmental disorders, reflecting specific deficits in the acquisition of literacy skills and oral language, respectively [1]. For both disorders, a diagnosis is achieved by excluding known causes of the deficits, such as co-occurring sensory or neurological impairment or lack of educational opportunity. RD and SLI are complex traits resulting from the interaction of multiple factors of both genetic and environmental origin, yet the biological underpinnings and cascading cognitive deficits remain poorly understood. Several genes have been proposed as susceptibility candidates for both RD and SLI. The RD candidates include the *ROBO1*, *KIAA0319*, *DCDC2* and *DYX1C1* genes and the *MRPL19/C2ORF3* locus [2]. The SLI candidates include the *CMIP*, *ATP2C2* and *CNTNAP2* genes [3]. It has been shown that most of the dyslexia risk genes (*KIAA0319, DCDC2, DYX1C1* and *ROBO1*) are involved in cortical development and specifically in neuronal migration [4]. An important unanswered research question is how genes involved in such a general process as cortical development contribute to the risk for specific neurodevelopmental disorders. Several studies have addressed this research question by investigating whether candidate genes implicated in a specific disorder show pleiotropic effects, which could, for example, help explain the high comorbidity observed between SLI, RD and ADHD [5], [6], [7]. To date only modest associations have been reported for shared genetic factors across these separate conditions [8], [9], [10]. Recently we conducted an association study in the Avon Longitudinal Study of Parents and Children (ALSPAC) cohort to explore potential genetic overlaps between SLI and RD [11]. We analysed a panel of single nucleotide polymorphisms (SNPs), previously reported to be associated with either RD or SLI, and tested them for association with literacy and language related phenotypes. We reported highly specific effects for *DCDC2, KIAA0319* and *CMIP* with measures of single-word reading and spelling [11]. These data suggested genetic effects on specific and independent aspects of cognitive function rather than on multiple or more generalised phenotypes.

A direct genetic effect of the impact on broader cognitive abilities for RD and SLI candidate genes has never been tested. General cognitive ability, assessed with standardised intelligence tests, is a highly heritable trait [12], [13], yet very few genes have been identified that impact upon these trait [14]. Most of the reported candidate genes have lacked adequate sample size and replications [15]. A recent genome-wide association study (GWAS) supports the strong heritability of cognitive abilities, but with effects that are spread across a large number of genetic factors and therefore not easily detectable in isolation [16]. In the present study, we used a candidate gene approach to investigate a precise question, namely whether genetic risk factors for RD and SLI have a broader impact on general cognitive function. This hypothesis is supported by the consistent observation of significant correlation between reading abilities and general cognition. We analysed RD and SLI candidate genes for association with general cognitive ability in the ALSPAC cohort and detected a statistically significant association at the chromosome 2p12 dyslexia-associated locus. The effect size was small but reproducible in independent samples. Given previous associations between white matter structure and both language-related phenotypes [17] and IQ [18], we looked for any correlations between genotypes at the *MRPL19/C2ORF3* locus and white matter structure. We found associations in the posterior corpus callosum and cingulum, connecting large sections of the parietal, occipital and temporal cortices. The widespread connectivity of this white matter region is consistent with a more general effect on both language and intellectual function.

## Results

We analysed 19 SNPs for association with verbal IQ (VIQ) and non-verbal IQ (performance IQ; PIQ) in the ALSPAC child cohort (**Table 1**) across the *MRPL19/C2ORF3, KIAA0319, DCDC2, ATP2C2* and *CMIP* genes (**Table 2**). These markers were selected for previously reported associations with reading- and language-related phenotypes [11]. We detected significant associations (p-values<0.001) with VIQ and the SNPs rs714939 (*MRPL12/C2ORF3* locus) and rs6935076 (*KIAA0319*). The rs714939 SNP was also the only marker that showed a trend for association with PIQ (P = 0.006). A marker in *CMIP*, rs6564903, also showed a trend of association for VIQ (P = 0.004). Our previous study [11] showed that *KIAA0319* and *CMIP* are associated with a measure of single word reading (READ) [19] in a subset of the ALSPAC sample. Given the high correlation between VIQ and READ (r = 0.571; Table 1) [11], it is plausible that the association we observed here was driven by reading ability. Therefore, we included READ as covariate when analysing the association with VIQ (**Table 2**). This analysis showed that the associations with rs6935076 (*KIAA0319*) and rs6564903 (*CMIP*) became attenuated, whereas rs714939 remained significantly associated with VIQ. Therefore, while the association with the *KIAA0319* and *CMIP* markers may have been driven by the correlation with reading, the associations at the 2p12 locus appeared to be specific to IQ.

**Table 1:** Details of phenotypic measures.

	ALSPAC	SLI families	Dyslexia families	Dyslexia cases	Raine
VIQ (mean± SD)	WISC (109.1±16.3)	WISC (100.1±15.4)	BAS/WAIS (60.8±8)	BAS/WAIS (56.9±8)	PPVT (104.9±11.5)
PIQ (mean ± SD)	WISC (101.3±16.7)	WISC (95.4±16.7)	BAS (55±9)	BAS (55.5±7.4)	RCPM (61.8±25.8)
Age at test in years	8	6–16	6–25	8–18	10
Male∶female (ratio)	2,946∶2,959 (1.00)	319∶186 (1.70)	384∶254 (1.51)	196∶87 (2.25)	708∶683 (1.03)
VIQ∶READ correlation	0.57	0.62	0.40	0.44	0.40
PIQ∶READ correlation	0.35	0.37	0.32	0.41	0.20

VIQ (verbal IQ); PIQ (performance (non-verbal) IQ); SD (standard deviation); WISC (Wechsler Intelligence Scale for Children); BAS (British Abilities Scales); WAIS (Wechsler Adult Intelligence Scale); PPVT (Peabody Picture Vocabulary Test); RCPM (Raven's Coloured Progressive Matrices).

**Table 2:** Single-SNP analyses in the ALSPAC cohort.

			Verbal IQ			Performance IQ			Verbal IQ with READ as a covariate		
Chr	Gene (locus)	SNP	N	Beta	P	N	Beta	P	N	Beta	P
2	*MRPL19/C2ORF3*	rs1000585	5565	−0.038	0.06859	5560	0.005	0.8475	5159	−0.037	0.03911
2	*MRPL19/C2ORF3*	rs917235	5796	−0.043	0.03136	5789	−0.01	0.6773	5330	−0.044	0.0102
2	*MRPL19/C2ORF3*	rs714939	5555	0.069	**0.00099**	5550	0.068	0.00631	5149	0.061	**0.00066**
6	*DCDC2*	rs793862	5636	−0.034	0.148	5627	−0.018	0.5077	5182	−0.017	0.3908
6	*DCDC2*	rs807701	5837	−0.009	0.6705	5831	0.02	0.4176	5369	−0.003	0.8591
6	*DCDC2*	rs807724	5671	−0.026	0.2885	5664	−0.024	0.4166	5210	−0.013	0.5418
6	*DCDC2*	rs1087266	5850	−0.006	0.7773	5843	0.003	0.8914	5380	−0.001	0.9315
6	*KIAA0319*	rs761100	5825	−0.046	0.02341	5816	0.004	0.8837	5362	−0.045	0.00871
6	*KIAA0319*	rs6935076	5468	0.083	**0.00011**	5463	0.026	0.3026	5071	0.048	0.00862
6	*KIAA0319*	rs2038137	5569	−0.052	0.01319	5564	−0.015	0.5587	5161	−0.045	0.01142
6	*KIAA0319*	rs9461045	5641	−0.044	0.09869	5634	−0.013	0.6763	5190	0.002	0.9172
6	*KIAA0319*	rs2143340	5554	−0.053	0.06262	5549	−0.011	0.7384	5148	0.001	0.9517
16	*CMIP*	rs12927866	5570	−0.053	0.01085	5565	−0.017	0.4918	5163	−0.038	0.02997
16	*CMIP*	rs6564903	5750	−0.058	0.00385	5743	−0.024	0.3224	5289	−0.036	0.03382
16	*CMIP*	rs4265801	5565	0.028	0.1667	5560	0.013	0.5895	5159	0.02	0.2499
16	*CMIP*	rs16955705	5563	−0.035	0.0867	5558	0.001	0.9578	5158	−0.022	0.2104
16	*ATP2C2*	rs16973771	5503	0	0.985	5499	0.016	0.5133	5100	0	0.995
16	*ATP2C2*	rs2875891	5569	−0.007	0.728	5564	0.014	0.593	5161	−0.005	0.7846
16	*ATP2C2*	rs8045507	5560	−0.009	0.6748	5555	0.018	0.4787	5153	−0.003	0.8716

p-values<0.001 are emboldened.

BETA are relative to the minor allele.

Another SNP at the *MRPL19/C2ORF3* locus, rs917235, also showed a trend of association with VIQ (**Table 2**). For both rs917235 and rs714939, the G alleles are associated with lower performance. These were the same alleles associated with dyslexia in the original report [20]. The most robust association in this previous study was reported for two haplotypes, in different populations, that overlapped for rs917235 and rs714939; specifically, the “GG” haplotype formed by these two markers increased susceptibility to dyslexia. Accordingly, we also tested the rs917235/rs714939 haplotypes for association with VIQ (**Table 3**) in the ALSPAC cohort. The “GG” haplotype gave the strongest association with lower VIQ scores (P = 0.00009). When READ was used as a covariate, the association became weaker but remained significant (P = 0.00024). Furthermore, the “AA” haplotype showed significant association with higher VIQ scores and with an effect size of similar magnitude.

**Table 3:** Summary results for rs917235/rs714939 haplotype associations with VIQ in the ALSPAC cohort.

	N = 5428	Haplotype		Haplotype analysis with READ as a covariate	
		N = 5428		N = 5030	
HAPLOTYPE	%	BETA	P	BETA	P
GG	0.317	−0.093	**0.00009**	−0.074	**0.00024**
AG	0.299	0.004	0.857	−0.005	0.821
AA	0.236	0.063	0.0175	0.072	**0.00116**
GA	0.148	0.076	0.0231	0.038	0.176

p-values statistically significant are in bold.

To replicate these results, we tested the *MRPL19/C2ORF3* markers for association with VIQ and PIQ in four independent samples (**Table 4**). We consistently found a trend of association across the four collections, where either of the two originally associated markers had the G allele associated with lower performance. Associations were particularly evident in the SLI families (rs714939; P = 0.009; PIQ) and in the unrelated cases with dyslexia (rs917235; P = 0.0004; VIQ). The same SNPs did not show association with single word reading measures in previous studies using the same samples [9], [11], [21] excluding the possibility that these associations are driven by the high correlation between VIQ and reading ability. We also re-analysed all of the samples for association with reading using IQ as covariate, but again none of the samples including the ALSPAC cohort showed any associations (data not shown). The unrelated cases with dyslexia was the only sample that showed stronger association with VIQ, while the other samples showed associations mainly with PIQ. The dyslexia families and Raine samples only showed a modest trend of association, but it was in the same allelic direction (rs917235; P = 0.064 and rs714939; P = 0.059, respectively). Haplotype analysis in all replication sets revealed a similar trend, with the “GG” haplotype associated with lower performance, but did not show associations stronger than single SNP analyses (data not shown).

**Table 4:** P-values from single-SNP analyses for VIQ and PIQ in replication samples.

	ALSPAC			SLI families			Dyslexic families			Dyslexic cases			Raine		
SNP	N	VIQ	PIQ	N	VIQ	PIQ	N	VIQ	PIQ	N	VIQ	PIQ	N	VIQ	PIQ
rs1000585	5565	0.069	0.848	411 sib	0.393	0.272	604 sib	0.796	0.397	279	0.011	0.745	NT		
rs917235	5795	0.031	0.677	418 sib	0.574	0.304	599 sib	0.416	0.064	282	**0.0004**	0.464	1094	0.335	0.955
rs714939	5555	**0.001**	0.006	413 sib	0.034	0.009	592 sib	0.928	0.567	277	0.639	0.079	1080	0.846	0.059

p-values statistically significant are in bold.

RA = Risk alleles are given only for results showing trend of association (p<0.1); NT = not tested.

Risk allele was G for all SNPs in all samples.

**Figure 1:**
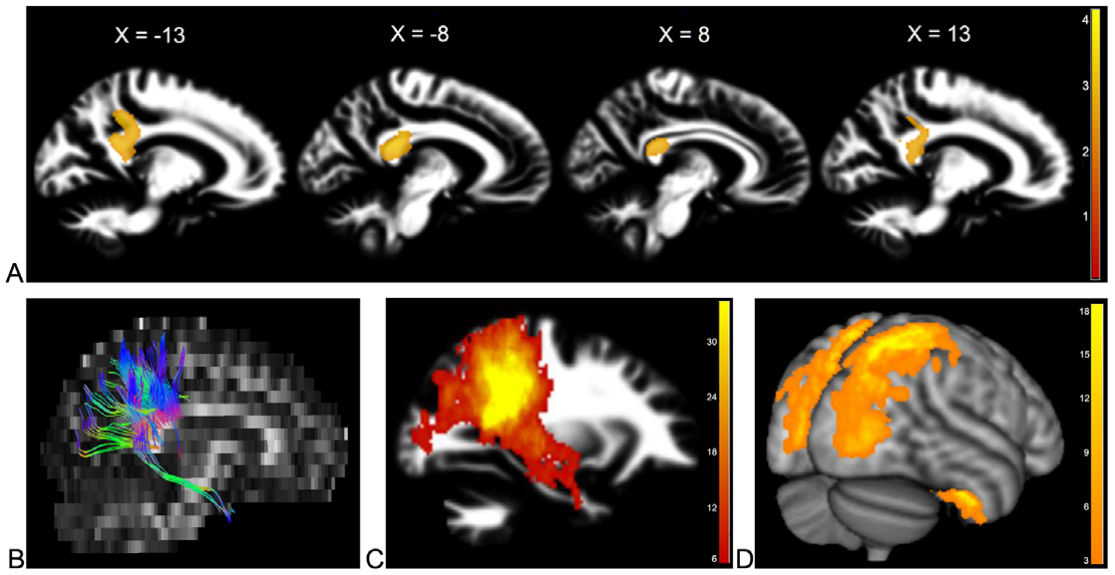
Imaging results. (A) Main association of rs917235 with white matter volume. Sagittal view of the significant cluster associated with rs917235 at the MNI coordinates of X = −13, −8, 8, and 13 mm relative to the midline. The background image is the average of all individuals' white matter segmented images. The colour-bar shows the z-scores of the statistical analysis. (B–D) Structural connectivity. (B) A sample of fiber tracking from one individual passing through the region associated with rs917235. Different colours show the direction of pathways (red for left-right, blue for superior-inferior, and green for anterior-posterior). (C) Overlay of tract tracing from 30 randomly selected individuals. The colour-bar is a count for the number of subjects; yellow shows the most probable pathways. (D) Cortical end-points of the white matter pathways showing that the fibers connect right postcentral gyrus, superior parietal lobule, precuneous, occipital cortex and temporal fusiform gyrus to the analogous left regions.

We assessed the effect of genetic variants at the *MRPL19/C2ORF3* locus on white matter structure in the Swedish cohort. Among seven genotyped markers, only rs917235 showed significant association with variation in white matter volume (P_corrected_ = 1.27×10^−3^ at cluster level with the threshold of P<0.01). Specifically, the G allele, already shown to be associated with lower IQ, was associated with lower white matter volume. This white matter cluster was located bilaterally and confined mainly to the posterior part of the corpus callosum and the cingulum (**Figure 1A**).

The significant cluster in the posterior corpus callosum (**Figure 1A**) was then used as a seed region for fiber tracking (see **Figure 1B** for tracking in one individual) performed in 30 randomly selected subjects. The pathways from each subject were then overlaid on a common template to identify the most consistent localization of pathways (**Figure 1C**). Next, we identified the parts of cortex that were closest to the end-points of the white matter tracts. This analysis showed that the pathways passing through the region connected the right postcentral gyrus, superior parietal lobule, precuneous, lateral occipital cortex and fusiform gyrus to analogous areas in the left hemisphere (**Figure 1D**).

## Discussion

We evaluated the effect of candidate genes for RD and SLI on measures of general cognitive ability in the ALSPAC cohort. The *MRPL19/C2ORF3* locus showed a statistically significant association with general cognitive abilities (VIQ and PIQ). *KIAA0319* and *CMIP* variants also showed associations, but these genes have previously been found associated with reading ability in the ALSPAC sample [11], hence the associations detected here could be an artefact due to the correlation between VIQ and measures of single word-reading. Indeed, the associations between the *KIAA0319* and *CMIP* markers and VIQ were attenuated when a reading measure was used as a covariate. Conversely, *MRPL19/C2ORF3* markers did not show any association with single-word reading in the ALSPAC cohort [11]. Furthermore, this association remained significant when reading ability was used as a covariate and is supported by a consistent trend of association with IQ in four independent samples. The association would not stand a genome-wide correction for significance level, but this would be consistent with a recent GWAS that predicts multiple and small-size factors contributing to IQ [16]. Our candidate gene approach was based on the selection of previously reported disorder-associated genotypes, suggesting a more generalised effect across multiple cognitive traits. Our interpretation is that the chromosome 2 locus has an effect on cognition by influencing neurodevelopment. The effect on specific endophenotypes will depend on ascertainment criteria, methodology for phenotypic assessment and differences in genetic background. All these elements will have a significant role in the outcome of association analyses when sample sizes are relatively small.

To date, only a few common genetic variants have been associated with measures of verbal and nonverbal reasoning. These include variants in the *CHRM2, COMT* and *BDNF* genes [14]. GWASs, which have recently had an enormous success in identifying genetic variants contributing to complex traits, had little success in mapping variants associated with cognitive abilities, with no indication of major genetic contributing loci [16], [22], [23]. The generally accepted model proposes that cognitive abilities are influenced by many genes of small effect [24] and are therefore difficult to map in the relatively small-sized samples currently available. Gene group-base analysis might be more effective by testing biological functions determined by multiple genes [25].

Both rs714939 and rs917235 are within an intergenic region between the *FAM176A* and *MRPL19/C2ORF3* loci. The original study associating this locus with dyslexia showed an effect on gene expression of the co-regulated genes *MRPL19* and *C2ORF3*, and differential expression across a set of brain regions [20]. These genes are transcribed in a head-to-head orientation on chromosome 2p12. Visualisation of *MRPL19* and *C2ORF3* in the Allen Brain Atlas (a resource of human gene expression data derived from 3 post-mortem males aged 24 to 57 years old at time of death; http://www.brain-map.org/), show that these two genes are most highly expressed in white matter of the corpus callosum and the cingulum when compared to all other regions. This is in contrast to *KIAA0319*, *CMIP* and *ATP2C2* which show very low expression in white matter, and *FAM176A* which shows moderate expression. There is no direct evidence that these two SNPs are the causative variants and it is likely that together they tag the effect of a nearby functional factor. The *FAM176A* gene, also expressed in fetal brain, cannot be ruled out as being influenced by these two SNPs. Interestingly, rs714939 is located in a region of high H3K4Me1 marks (ENCODE ChIP-Seq data, GRCh37/hg19 assembly visualised in the UCSC Genome Browser (http://genome.ucsc.edu/cgi-bin/hgGateway)). H3K4Me1 is a monomethylation of lysin 4 of the H3 histone protein and this modification is often found near regulatory elements. This region of high H3K4Me1 spans 9–10 kb and could be involved in the cis-regulation of any of the three neighbouring genes.

The imaging results provide a neural correlate to the genetic polymorphism. Our interpretation is that the genetic variants contribute to structural variation in a relatively large area of white matter which affects both reasoning and reading. It should be pointed out, however, that this is an association study that does not provide any direction of causality. It cannot be excluded that genes affect behavior, which in turn affect the white matter. Our neuroimaging analysis revealed a significant association between rs917235 and white matter volume of the posterior part of the corpus callosum and cingulum. The G allele, associated with lower IQ in the behavioural analysis, was specifically associated with lower white matter volume in this region. Previous studies have reported correlations between white matter structure and measures of IQ [26], [27], [28], [29], supporting the idea that neural connectivity of the brain is an endophenotype underpinning general intellectual ability [18]. Tract tracing of fibers passing through the correlated white matter region showed that they establish long-range connections with large regions of the parietal and occipital cortices, and a smaller region within temporo-occipital cortex. Intra- and superior parietal have been associated with performance on reasoning tasks [30] and the inferior parietal cortex is important for language-related functions [31]. The thickness of the splenium of the corpus callosum, which connects large parts of the occipital, parietal and temporal lobes, has been previously associated with intelligence [32]. Based on parieto-frontal integration theory of intelligence (P-FIT) [30], the lateral cortex and fusiform gyrus from the temporal lobe are involved in cognitive ability since they participate in visual perception, recognition and imagination. The superior parietal, supramarginal and angular gyri are involved in structural symbolism and abstraction. These regions are also important for language-related functions. The posterior part of the corpus callosum has been previously reported as a white matter region with structural differences between normal and dyslexic readers. [33], [34], [35], [36]. It has been also shown that the posterior part of the corpus callosum is bigger in children with dyslexia rather than in typically developing children [37] The widespread connectivity of the white matter region associated with rs917235 is thus consistent with the previous neuroimaging associations to both language and general cognitive abilities. However, substantially more cortical regions and associated brain functions are likely to underlie general cognitive abilities. The integration of genetics with structural and functional imaging approaches holds potential for further elucidating the effects of genes on the normal and atypical development of cognitive function. With a similar approach we showed that three dyslexia susceptibility genes *DYX1C1, DCDC2* and *KIAA0319* are associated with white matter volume in distinct but overlapping regions of the left temporo-parietal hemisphere, and that the white matter volume in these regions also correlated with reading ability [38]. A recent study found association between candidate genes for language and reading impairment (*FOXP2* and *KIAA0319*, respectively) and regionally specific brain activations assessed with fMRI [39]. Another study reported two independent SNP associations, both on chromosome 12, with brain volume measures as well as suggestive evidence of an effect on cognitive abilities [40]. Most recently, a study has found a genome-wide significant association between a SNP (rs2298948) within *C2ORF3* (called *GCFC2* in that study) and hippocampal volume [41]. The hippocampus plays an important role in learning and spatial memory, and this is correlated with hippocampus volume.

The association of cognitive abilities with a genetic locus, originally identified as a candidate for dyslexia susceptibility, is independent from a clinical diagnosis of dyslexia and SLI. The association was statistically significant in the ALSPAC sample, which represents the general population, and we did not observe any associations with reading-related measures in either ALSPAC or the other replication samples, including the ones selected for dyslexia or SLI. It is possible that the original association with developmental dyslexia at the *MRPL19/C2ORF3* locus [20] was due to a sampling effect and the high correlation between the verbal component of cognitive abilities and reading. Alternatively, the same genetic variants may have different phenotypic effects when combined with alternative genetic or environmental factors and would become apparent in separate sample subgroups. Multivariate genetic analysis have consistently suggested a correlation of about 0.6 between general cognitive ability and learning [42] but there is less agreement in estimating the effects across the range of the entire phenotypic distribution [43]. This would imply that some factors may have specific effects only at the extremes of the phenotype where disorder diagnosis would apply.

In summary, we report an association of measures of general cognitive abilities with the chromosome 2p12 locus implicated in dyslexia. We show, for the first time, that the same genetic locus is associated with white matter volume in the posterior corpus callosum. Furthermore, fibers throughout this region connected cortical regions involved in both language and general cognitive abilities. Follow-up studies might identify the functional genetic variant(s) and the gene(s) implicated. Such findings will contribute to our understanding of the biological pathways underlying normal and atypical cognition and the possible shared factor(s) mediating general cognitive functions and highly prevalent developmental disorders such as dyslexia.

## Materials and Methods

### Ethics Statement

Informed written consent was obtained from the parents, with the option for them or their children to withdraw at any time. Ethical approval for the ALSPAC cohort was obtained from the ALSPAC Law and Ethics Committee and the Local Research Ethics Committees. Ethical approval for the SLIC cohort was granted by local ethics committees. Ethical approval for the Oxford/Reading and Aston studies was acquired from the Oxfordshire Psychiatric Research Ethics Committee (OPREC O01.02). For the Raine Study, participant recruitment and all follow-ups of the study families were approved by the Human Ethics Committee at King Edward Memorial Hospital and/or Princess Margaret Hospital for Children in Perth. For the Swedish sample, the study was approved by the Ethics Board of the Karolinska University Hospital (Stockholm).

### Initial Sample

The ALSPAC cohort consists of over 14,000 children from the southwest of England that had expected dates of delivery between 1^st^ April 1991 and 31^st^ December 1992 [44]. From age 7 years, all children were annually assessed for a wide range of physical, behavioural, and neuropsychological traits, including reading and language-related measures. DNA is available for approximately 11,000 children. For this study, individuals with a non-white ancestry were excluded and after filtering for missing genotypic or phenotypic data we conducted the analysis in a sample of 5905 children. Cognitive ability was assessed using the Wechsler Intelligence Scales for Children (WISC-III) [45] for both verbal and performance IQ (VIQ and PIQ, respectively; **Table 1**). The VIQ scale included the subtests for Information, Similarities, Arithmetic, Vocabulary, Comprehension and Digit span. The PIQ scale included the subtests for Picture completion, Coding, Picture arrangement, Block design and Object assembly.

### Replication Samples

The SLI Consortium (SLIC) cohort has been described in detail previously [46], [47], [48]. This family-based sample includes approximately 400 individuals from 181 families. The samples were assessed at one of five separate centres across the UK: The Newcomen Centre at Guy's Hospital, London, the Cambridge Language and Speech Project (CLASP [49]), the Child Life and Health Department at the University of Edinburgh [50], the Department of Child Health at the University of Aberdeen and the Manchester Language Study [51], [52]. Cognitive ability of all children in the SLIC sample was assessed using the WISC-III [45] applying the same VIQ and PIQ subtests listed for ALSPAC. In the SLIC sample, the PIQ score (cut-off at PIQ>80) was used to exclude children whose language problems were accompanied by deficits in non-verbal skills.

The dyslexia-based sample has been described previously [53], [54]. It includes 684 siblings from 288 unrelated nuclear families and 282 unrelated cases with dyslexia, recruited through the Dyslexia Research Centre clinics in Oxford and Reading, and the Aston Dyslexia and Development Assessment Centre in Birmingham. VIQ and PIQ were obtained from the BAS similarities and BAS matrices subtests respectively [55]. The similarities sub-scale of the Wechsler Adult Intelligence Scales (WAIS), a measure analogous to the BAS similarities test, was used when age was >17.5 years [45].

The Western Australian Pregnancy Cohort (Raine) study is a longitudinal investigation of 2900 pregnant women and their offspring recruited between 1989 and 1991 [56]. From the original cohort, 2868 children have been followed over two decades. The Raine sample is representative of the larger Australian population (88% Caucasian); only those children with both biological parents of white European origin were included in the current analyses. Verbal and non-verbal ability was assessed at 10 years of age using the Peabody Picture Vocabulary Test–Revised (PPVT-R) [57] and the Raven's Coloured Progressive Matrices (RCPM) [58], respectively. The PPVT-R provides a measure of receptive vocabulary, requiring children to select which of four pictures corresponds to an aurally presented word. Raw scores are converted to a Verbal IQ, standardized for age 2 years and above (based around a mean of 100 and a SD of 15). RCPM is a 36 item multiple choice test that presents a matrix-like arrangement of figural symbols and requires the child to select the missing symbols from a set of six alternatives. Raw scores are converted to percentiles, which provide an indication of performance relative to other children of a similar age. This assessment is standardized for children between 4.9 and 12.0 years of age.

The Swedish sample used for neuroimaging consists of 76 Swedish speaking children and young adults (age range 6 to 25 years) randomly selected from the “Brainchild” study, a longitudinal study of typical development [59], [60]. The participants were from the population register in the town of Nynäshamn, Sweden, and showed no evidence of neurological or psychological disorders. DNA was available for all subjects.

### Genotyping and statistical analysis

We analysed 19 SNPs across the *MRPL19/C2ORF3, KIAA0319, DCDC2, ATP2C2* and *CMIP* loci that were recently genotyped in the ALSPAC child cohort [11]. The sample size in the present study is larger because we did not apply an IQ filter as described in the previous analysis. SNPs were genotyped using either Sequenom iPLEX assays according to the manufacturer's instructions or the KBiosciences service using their in-house technology (http://www.kbioscience.co.uk/). The genotyping in the samples selected for dyslexia and SLI was conducted with Sequenom iPLEX assays as part of previous studies [9].

For the Raine study, DNA samples were genotyped on an Illumina 660 Quad Array [61].

For the Swedish sample, seven SNPs (rs3088180, rs4853169, rs917235, rs6732511, rs714939, rs17689640 and rs17689863) located at the *MRPL19/C2ORF3* locus were genotyped using the Sequenom iPLEX system as previously described [59].

Quantitative analyses were performed within PLINK (1.07) [62] using additive tests of association. We included two additional phenotypes (VIQ and PIQ) to the multiple testing correction applied in our previous study of the ALSPAC sample [11] which corrected for the analysis of 11 clusters of SNPs showing significant linkage disequilibrium (LD; r^2^>0.6) and 2 phenotypes. Therefore we corrected here the significance level of P = 0.05 for 44 independent tests (11 SNP clusters analysed for four phenotypes) resulting in P = 0.001. The ALSPAC cohort has been tested previously for other SNPs and phenotypes, therefore these tests should be considered in calculating a significant threshold p-value, or the genome-significant threshold of 5×10^−8^ should be applied. However, this would be too conservative for the scope of this study which analyses previously reported genetic markers and tests a specific hypothesis rather than conducting an explorative exercise. Family-based cohorts were analysed using QTDT [63].

### Image analysis

For the Swedish sample, three-dimensional structural T1-weighted imaging with magnetization-prepared rapid gradient echo sequence (TR = 2300 ms, TE = 2.92 ms, 256×256 mm, 176 sagittal slices and 1 mm^3^ voxel size) with the field of view of 256×256 mm, 256 slices, and 1 mm^3^ voxel size was carried out on all the participants and repeated two years later for 69 subjects. White matter segmentation, followed by an alignment technique, was performed on the structural data using the Diffeomorphic Anatomical Registration using Exponentiated Lie algebra (DARTEL) toolbox in SPM (www.fil.ion.ucl.ac.uk/spm/software/spm5). Images were then spatially smoothed with an 8 mm Gaussian kernel. The DARTEL outputs are white matter segmented images which reflect the signal intensity modulated by volume transformations applied to individual images to register them into the MNI template.

Diffusion tensor imaging (DTI) was also acquired using a field of view 230×230 mm, matrix size 128×128 mm, 19 slices with 6.5 mm thickness b-max 1000 s/mm^2^ in 20 directions. Eddy current and head motion were corrected using affine registration to a reference volume using FSL (www.fmrib.ox.ac.uk/fsl/). The diffusion tensors were then computed for each voxel and the DTI and fractional anisotropy (FA) data were then constructed.

The seven SNPs were entered separately as a main factor in a flexible factorial design second-level SPM analysis. This included both the individual images with and without repeated measures, to assess the variation of white matter with respect to the genetic markers and was corrected for the effect of age, sex, handedness and total white matter volume. Age X gene and gender X gene interaction effects were also included in the model. As a part of this exploratory analysis, the significance level was corrected at the cluster level using non-stationary cluster extent correction [64] for multiple comparisons resulting from searching the entire white matter volume as well as for the seven SNPs (Bonferroni correction, P_corrected_<0.0014 for comparison of searching the entire brain with a threshold of P<0.01, as implemented in the SPM software).

The region showing the significant effect was saved as a binary region of interest (ROI). This ROI was then transformed to each individual's DTI space, to be used as a seed ROI for white matter fiber tracking. Deterministic fiber tracking was applied on 30 randomly selected subjects by starting tractography from the ROI following the principal eigenvector direction using 1 mm steps, considering thresholds of 0.15 for FA values and 30 for angular degree using ExploreDTI v4.7.3. (www.exploredti.com). Computed tracts from all individuals were then averaged across all 30 participants to derive a probabilistic map of the white matter pathways passing through the overlapping areas.
